# Clinical significance of serum CXCL9, CXCL10, and CXCL11 in patients with lupus nephritis

**DOI:** 10.1002/iid3.1368

**Published:** 2024-08-22

**Authors:** Shuo Wang, Yanhui Cui

**Affiliations:** ^1^ Department of Nephrology Zibo Central Hospital Zibo China; ^2^ Department of Rheumatism Immunity Zibo Central Hospital Zibo China

**Keywords:** CXCL9, CXCL10, CXCL11, lupus nephritis, systemic lupus erythematosus

## Abstract

**Study Design:**

Lupus nephritis (LN) is an autoimmune disease as a complication of systemic lupus erythematosus (SLE). LN is typically diagnosed through a combination of clinical evaluation as index scoring, and kidney biopsy as a more accurate but invasive examination. In the current study, we assessed serological markers including IFN‐γ‐inducible chemokines C‐X‐C motif chemokine ligand (CXCL)9, CXCL10, and CXCL11 in diagnosing LN.

**Methods:**

A retrospective analysis was conducted on 160 SLE patients with and without LN. Fasting venous blood was collected from the study subjects for measuring serum levels of CXCL9, CXCL10, and CXCL11. The assessment of clinical disease activity in SLE was conducted using the SLE Disease Activity Index (SLEDAI)‐2000 scoring system. LN disease activity was conducted using the Austin scoring system. LN was further confirmed following kidney biopsy, and data were compared by receiver operating characteristic (ROC) analysis.

**Results:**

SLE patients with LN showed longer SLE duration, enhanced SLEDAI scores, lower serum anti‐ds‐DNA antibody levels when compared to SLE patients without LN. Specifically, these patients had significantly higher serum levels of CXCL9, CXCL10 and CXCL11. CXCL9, CXCL10, and CXCL11 showed positive correlation with SLE disease activity in SLE patients with LN. ROC analysis of CXCL9, CXCL10, and CXCL11 showed substantial enhancement of sensitivity and specificity for the diagnosis of LN in the patients with SLE.

**Conclusions:**

Serum CXCL9, CXCL10, and CXCL11 levels may improve the sensitivity and specificity for the diagnosis of LN in SLE patients.

## Graphical abstract

1. Receiver operating characteristic analysis of C‐X‐C motif chemokine ligand (CXCL)9, CXCL10, and CXCL11 showed substantial enhancement of sensitivity and specificity for the diagnosis of lupus nephritis. 2. Serum CXCL9, CXCL10, and CXCL11 levels can significantly improve sensitivity.

## 1 INTRODUCTION

Our current work involves clinical work on lupus nephritis (LN), which is a complication of systemic lupus erythematosus (SLE).^1^ SLE^2^, ^3^ is a common chronic autoimmune disease that often affects women of childbearing age.^4^ It can affect multiple organ systems and presents with various clinical manifestations. LN is a common and severe complication of SLE, with up to 50% of adult SLE patients experiencing varying degrees of kidney damage during the course of the disease. Thus, it is an important factor that affects the prognosis of SLE patients.

Currently, in clinical practice, the assessment of disease activity in LN has primarily relied on disease activity index (AI) scoring. However, it is often challenging to determine whether positive indicators are caused by LN. Kidney biopsy, as an invasive examination, imposes significant trauma and risks to patients, and it cannot be repeated, thus having significant limitations. Serological markers, on the other hand, play a crucial role in diagnosing and differentiating various diseases in clinical practice due to their simplicity, speed, and high specificity.

IFN‐γ is a type of pro‐inflammatory cytokine primarily secreted by Th1 cells. It plays a crucial role in the pathogenesis of SLE and is closely associated with kidney damage in SLE.^5^, ^6^, ^7^ Chemokines are a group of structurally similar, low‐molecular‐weight cytokines with molecular weights ranging from approximately 8–14 kD. They play a role in attracting white blood cells to sites of infection or inflammation. C‐X‐C motif chemokine ligand (CXCL)9, CXCL10, and CXCL11 are all IFN‐γ‐inducible CXC chemokines and are efficient lymphocyte chemo‐attractants.^8^, ^9^ Their common receptor, CXCR3, is found on the surface of various immune cells, including NK cells, plasma‐like cells, dendritic cells, and especially activated T cells. CXCL9, CXCL10, CXCL11, and their receptor CXCR3 are associated with the pathogenesis of several autoimmune diseases, including organ‐specific autoimmune diseases like type‐1 diabetes, hyperthyroidism, Graves' disease, as well as systemic autoimmune diseases such as rheumatoid arthritis, SLE, multiple sclerosis, and Sjögren's syndrome.^10^, ^11^, ^12^

Herein, the aim of this study is to investigate the diagnostic value of serum CXCL9, CXCL10, and CXCL11 in LN and their correlation with the activity level of LN.

## 2 MATERIALS AND METHODS

### 2.1 Participants

This is a retrospective study and was approved by Zibo Central Hospital (#2023.05.284). Informed written consent from the participants was waived. We did retrospective analysis on 160 cases of patients with SLE treated in our department over a 3‐year period. This study reviewed the cases from 2018 to 2020. Based on whether SLE patients had concurrent LN, they were divided into the SLE‐LN group (*n* = 68) and the non‐LN group (SLE, *n* = 92). LN was further confirmed by the kidney biopsy.

#### 2.1.1 Inclusion criteria

Patients with SLE were accurately diagnosed based on the classification criteria recommended by the American College of Rheumatology (ACR) in 1997.^13^ Patients were aged 18 years or older. Clinical data for the patients were complete.

#### 2.1.2 Exclusion criteria

Patients with significant organ pathologies such as cardiovascular diseases, hepatitis, and other kidney diseases were excluded. Pregnant and lactating women were also excluded. Patients with non‐SLE associated blood system disorders, endocrine system disorders, or concurrent rheumatic diseases were excluded. Additionally, individuals with mixed connective tissue diseases, systemic sclerosis, and other autoimmune diseases, as well as those with malignancies, were excluded from the study.

70 healthy participants were selected who underwent medical examinations at our hospital during the same period as healthy controls (HC). The exclusion criteria for the HCs were consistent with those mentioned earlier, and they were not diagnosed with SLE.

### 2.2 Research methods

SLE patients were diagnosed with LN when they had any of the following clinical and laboratory abnormalities, including: (1) proteinuria persisting > 0.5 g/24 h, or urine protein+++ on random urine examination, or urine protein/creatinine ratio > 500 mg/g (50 mg/mmol); (2) cellular casts including red blood cell casts, hemoglobin casts, granular casts, tubular casts, or mixed casts; (3) active urine sediment (excluding urinary tract infection, urine white blood cells > 5/HPF, urine red blood cells > 5/HPF), or red blood cell casts, or white blood cell casts. Based on the above criteria, further kidney biopsy was performed for confirmation of LN. The indication for kidney biopsy is when any of the above SLE patients have abnormal laboratory tests. LN was confirmed by the kidney biopsy.

10 mL of fasting venous blood was collected from the subjects and preserved before use. After centrifugation (3000 rpm, 15 min), serum was collected and stored at −80°C. The concentrations of serum CXCL9, CXCL10, and CXCL11 were detected using ELISA kits purchased from R&D Biosystems.

The assessment of LN disease activity was conducted using the Austin scoring system, which evaluates the activity of kidney involvement in LN patients. It is represented by the AI and includes the following six components, each scored from 0 to 3 points: glomerular endothelial proliferation, neutrophil infiltration/rupture, subendothelial deposits, interstitial inflammation cell infiltration, fibrinoid necrosis, and cellular crescents. The total score ranges from 0 to 24 points. Based on the AI score, LN patients were divided into the LN active group (AI score ≥ 6 points) and the LN non‐active group (AI score < 6 points).

### 2.3 SLE clinical assessment

The assessment of clinical disease activity in SLE was conducted using the SLE Disease AI (SLEDAI‐2000) scoring system,^14^ established by the American Rheumatism Association (ARA). The scoring is as follows: Total score 0‐4: Indicates essentially no activity; Total score 5–9: Indicates mild activity; Total score 10–14: Indicates moderate activity; Total score ≥ 15: Indicates severe activity.

### 2.4 Statistical analysis

The sample size was calculated as *n* = (*Zα*/2 × *σ*/*E*)2, where n means sample size, *Zα*/2 means degree of confidence, σ means standard deviation and *E* means margin of error. Estimates of effect size and standard deviation were based on the existing literature and our previous experiences. To calculate the power of analysis we assumed *α* = .05 and *β* = .2. The estimated sample size was 150 to obtain a 95% confidence interval with a margin of error of 5%. The comparisons of data between the two group were done by Mann–Whitney test or Unpaired *t‐*test with Welch's correction or Fisher's exact test or Chi‐square test; Brown–Forsythe ANOVA test followed by Dunnett's T3 multiple comparisons test. The data presented are mean ± standard deviation (SD) or *n* (percentage). *p* < .05 was considered a statistically significant difference.

## 3 RESULTS

### 3.1 Demographic and clinical characteristics

In the current study, we conducted a retrospective analysis of 160 patients with SLE. The patients were divided into two groups based on whether they had concurrent LN: the SLE‐LN group (*n* = 68) and the non‐LN group (SLE, *n* = 92). Table 1 shows a comparison of clinical data between the two groups.

**Table 1:** Demographic and clinical factors for lupus nephritis (LN) among patients with systemic lupus erythematosus (SLE).

	SLE (*n* = 92)	SLE‐LN (*n* = 68)	*p*‐Value
Age (years)	36.39 ± 9.15	37.82 ± 9.84	.281
Body mass index (kg/m^2^)	23.58 ± 3.73	24.17 ± 4.06	.325
Gender			
Male	9 (9.8%)	8 (11.8%)	.797
Female	83 (90.2%)	60 (88.2%)	
Duration of SLE (years)	3.83 ± 2.17	4.93 ± 2.43	.003
Hypertension			
Yes	28 (30.4%)	25 (36.8%)	.497
No	64 (69.6%)	43 (63.2%)	
Diabetes mellitus			
Yes	16 (17.4%)	19 (27.9%)	.125
No	76 (82.6%)	49 (72.1%)	
SLEDAI			
0–4	12 (13.1%)	0 (0%)	<.001
5–9	37 (40.2%)	9 (13.2%)	
10–14	34 (36.9%)	33 (48.5%)	
≥15	9 (9.8%)	26 (38.2%)	
Serum anti‐ds‐DNA antibody			
Positive	37 (40.2%)	52 (76.5%)	<.001
Negative	55 (59.8%)	16 (23.5%)	
Austin activity index			
LN active	‐	24 (35.3%)	‐
LN inactive		44 (64.7%)	
Serum CXCL9 (pg/mL)	97.24 ± 28.29	130.88 ± 33.03	<.001
Serum CXCL10 (pg/mL)	98.36 ± 25.33	125.14 ± 30.45	<.001
Serum CXCL11 (pg/mL)	63.37 ± 20.23	94.11 ± 26.41	<.001

*Note*: The data presented are mean ± SD or *n* (percentage). The comparisons of data between the two groups were done by Mann–Whitney test or Unpaired t test with Welch's correction or Fisher's exact test or Chi‐square test.

Abbreviations: CXCL, C‐X‐C motif chemokine ligand; SLEDAI, systemic lupus erythematosus disease activity index.

The two groups of patients did not differ significantly in terms of age, gender, body mass index (BMI), or the presence of diabetes and hypertension. However, there were significant differences between the two groups in terms of SLE duration (3.83 ± 2.17 in SLE vs.4.93 ± 2.43 in SLE‐LN, *p* = .003), SLE Disease AI (SLEDAI) scores (9.8% of severe activity in SLE vs. 38.2% of severe activity in SLE‐LN, *p* < .001), serum anti‐ds‐DNA antibody levels (59.8% negative in SLE vs. 23.5% of negative in SLE‐LN, *p* < .001), as well as serum concentrations of CXCL9 (97.24 ± 28.29 in SLE vs. 130.88 ± 33.03 in SLE‐LN, *p* < .001), CXCL10 (98.36 ± 25.33 in SLE vs. 125.14 ± 30.45 in SLE‐LN, *p* < .001), and CXCL11 (63.37 ± 20.23 in SLE vs. 94.11 ± 26.41 in SLE‐LN, *p* < .001). Patients with concurrent LN had significantly higher serum levels of CXCL9, CXCL10, and CXCL11 compared to the non‐LN group (SLE). In addition, we further clarified whether SLEDAI in SLE patients was related to the levels of serum CXCL9 and CXCL10 with CXCL11 levels. It was found that there was significant positive correlation between SLEDAI in SLE patients and serum levels of CXCL9, CXCL10 or CXCL11 levels (Supporting InformationS1: Table S1), demonstrating that chemokine levels are positively correlated with the disease activity of SLE. We further separately analyzed the correlation between SLEDAI scores in the SLE group and serum CXCL9, CXCL10, and CXCL11 levels, reflecting the correlation between non‐renal SLEDAI scores and serum CXCL9, CXCL10, and CXCL11 levels. The results show that in SLE patients without LN, serum CXCL9 had a weak but significant positive correlation with SLEDAI, while the correlations of serum CXCL10 and CXCL11 with SLEDAI were not statistically significant (Supporting InformationS1: Table S2). These findings suggest that serum CXCL9, CXCL10, and CXCL11 have greater clinical significance for LN.

### 3.2 SLE‐LN patients show significantly higher serum CXCL9, CXCL10, and CXCL11 levels, and showed positive correlation with each other

**Figure 1:**
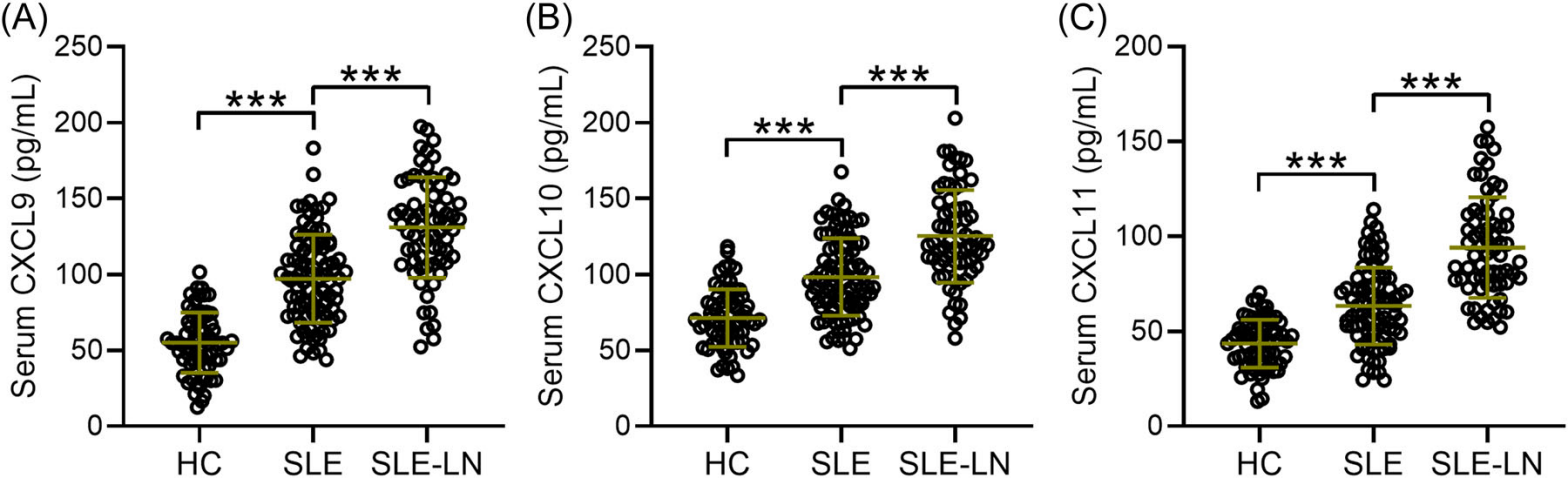
Comparisons of serum CXCL9 (A), CXCL10 (B), and CXCL11 (C) among systemic lupus erythematosus patients with lupus nephritis (SLE‐LN, *n* = 68) or not (SLE, *n* = 92) and healthy controls (HC, *n* = 70). Data were shown with mean ± SD. ***p < .001. Brown–Forsythe ANOVA test followed by Dunnett's T3 multiple comparisons test. CXCL, C‐X‐C motif chemokine ligand; SLE‐LN, systemic lupus erythematosus‐lupus nephritis.

Seventy healthy individuals who underwent medical examinations at our hospital during the same period were selected as HCs. The exclusion criteria for the HCs were consistent with those mentioned earlier, and they were not diagnosed with SLE. Comparison of the serum levels of CXCL9, CXCL10, and CXCL11 in 70 HCs, 92 SLE patients without concurrent LN, and 68 SLE patients with concurrent LN was shown in Figure 1. SLE‐LN patients showed significantly higher serum CXCL9 (*p* < .001, Figure 1A), CXCL10 (*p* < .001, Figure 1B), and CXCL11 (*p* < .001, Figure 1C) levels compared to SLE patients.

**Figure 2:**
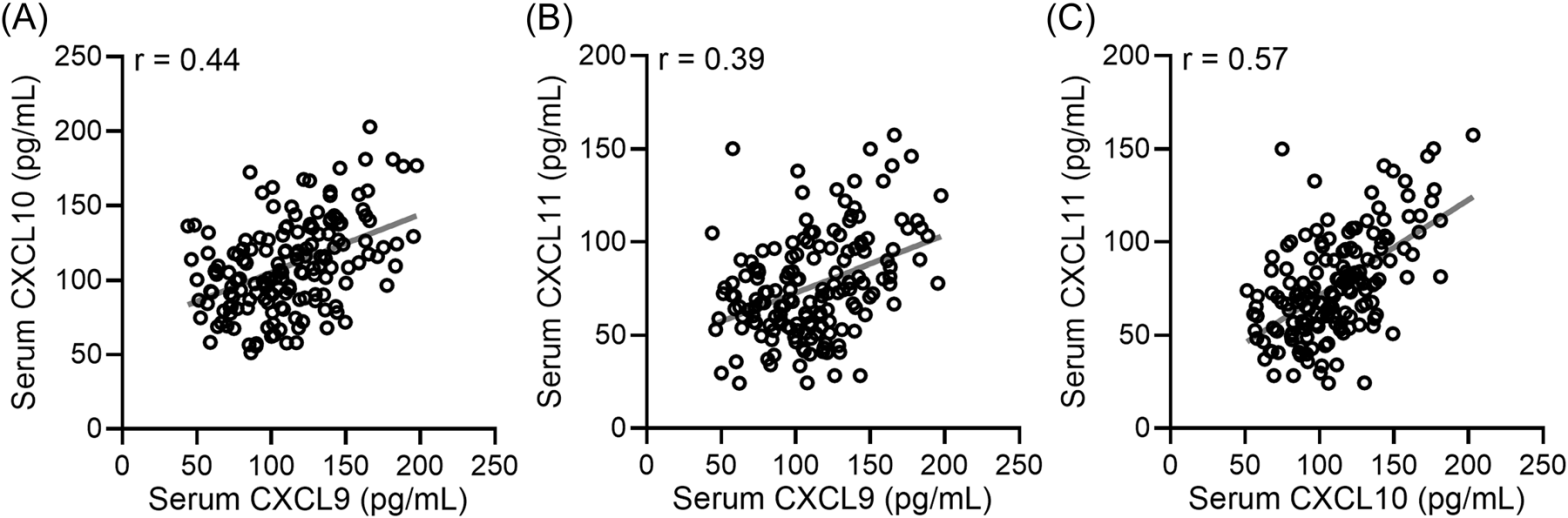
Pearson correlation analysis of serum CXCL9 with CXCL10 (A), CXCL9 with CXCL11 (B), CXCL10 with CXCL11 (C) in all the enrolled systemic lupus erythematosus patients (*n* = 160). *p* < .001 for all. CXCL, C‐X‐C motif chemokine ligand.

We further did an analysis of the correlation between serum levels of CXCL9 and CXCL10 (Figure 2A), CXCL9 and CXCL11 (Figure 2B), as well as CXCL10 and CXCL11 (Figure 2C) in 160 SLE patients, including those with and without concurrent LN. It was evident that there was a significant positive correlation between all three pairs of variables, as indicated by the correlation coefficients in the figure. All *p*‐values were less than 0.001 for CXCL10 versus CXCL9 (*r* = .44, Figure 2A), CXCL11 versus CXCL9 (*r* = .39, Figure 2B), CXCL11 versus CXCL10 (*r* = .57, Figure 2C).

### 3.3 Receiver operating characteristic (ROC) analysis showed positive correlation of CXCL9, CXCL10, CXCL11 with SLE patients with LN

**Figure 3:**
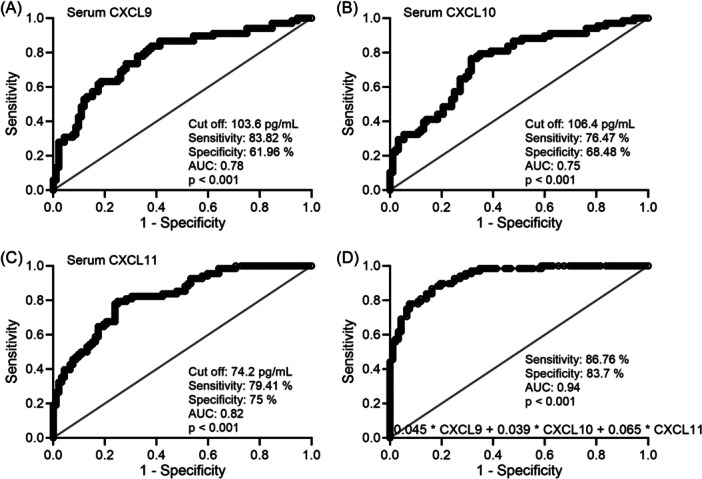
ROC analysis of serum CXCL9 (A), CXCL10 (B), CXCL11 (C), and their combined test model (D) for the diagnosis of lupus nephritis (LN) among patients with systemic lupus erythematosus (SLE). CXCL, C‐X‐C motif chemokine ligand; ROC, receiver operating characteristic.

We then did ROC analysis of the diagnostic value of serum levels of CXCL9, CXCL10, and CXCL11, as well as their combined detection, for the diagnosis of LN in SLE patients. In the combined detection model (0.045 × CXCL9 + 0.039 × CXCL10 + 0.065 × CXCL11), the cutoff value was determined based on the maximum Youden's index. The figure provided detailed data on sensitivity and specificity. It was evident that the combined detection model significantly improved the area under the ROC curve compared to the individual factor detection, resulting in a substantial enhancement of sensitivity and specificity for the diagnosis of LN in SLE patients (serum CXCL9 [sensitivity 83.82%, specificity 61.96%, Figure 3A], serum CXCL10 [sensitivity 76.47%, specificity 68.48%, Figure 3B], serum CXCL11 [sensitivity 79.41%, specificity 75%, Figure 3C], combined [sensitivity 86.76%, specificity 83.7%, Figure 3D]).

**Figure 4:**
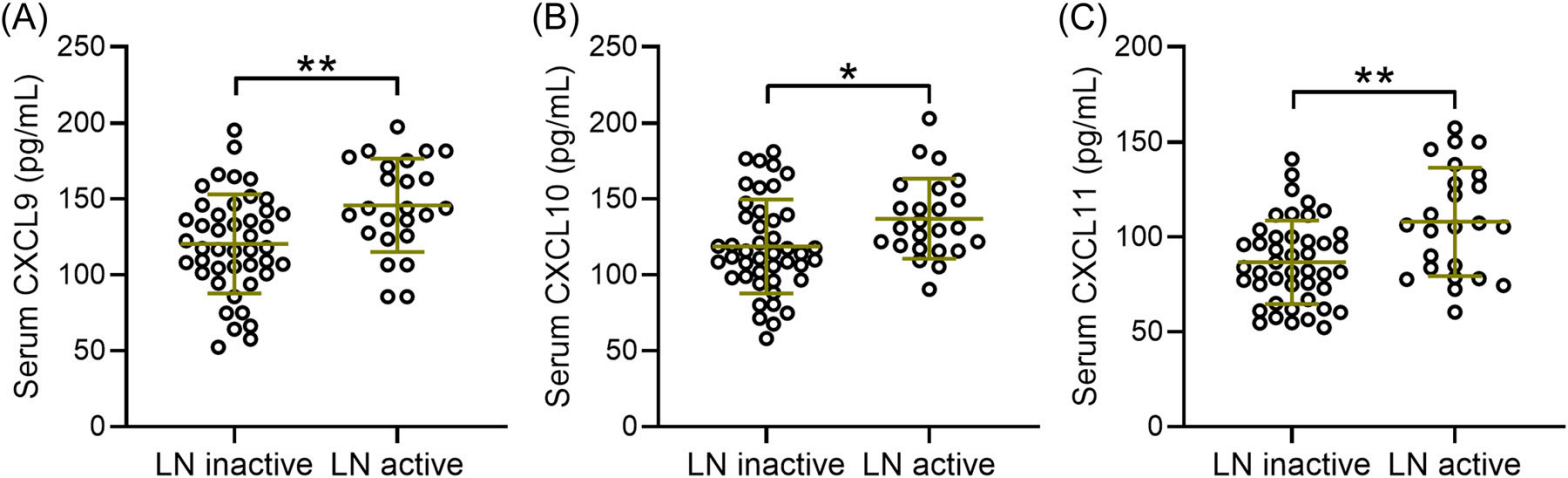
According to the Austin activity index, there were 44 inactive LN patients and 24 active LN patients. Comparisons of serum CXCL9 (A), CXCL10 (B), and CXCL11 (C) between inactive LN patients and active LN patients. *p < .05, **p < .01 from Unpaired *t‐*test with Welch's correction. CXC, C‐X‐C motif chemokine ligand; LN, lupus nephritis.

We analyzed the correlation between serum levels of CXCL9, CXCL10, and CXCL11 and the activity level of LN in SLE patients with concurrent LN. The assessment of LN disease activity was conducted using the Austin scoring system. LN patients were divided into the LN active group (AI score ≥ 6, totaling 24 cases) and the LN non‐active group (AI score < 6, totaling 44 cases). It was evident that patients in the LN active group had higher serum levels of CXCL9 (Figure 4A, 120.37 ± 32.65 pg/mL in LN inactive vs. 145.72 ± 30.68 pg/mL in LN active, *p* = .003), CXCL10 (Figure 4B, 118.61 ± 30.87 pg/mL in LN inactive vs. 137.04 ± 26.34 pg/mL in LN active, *p* = .013), and CXCL11 (Figure 4C, 86.59 ± 22.04 pg/mL in LN inactive vs. 107.86 ± 28.57 pg/mL in LN active, *p* = .004), indicating a correlation between the serum levels of CXCL9, CXCL10, and CXCL11 and the activity level of LN in SLE patients with concurrent LN.

## 4 DISCUSSION

SLE, is a chronic autoimmune disease that can affect various organs and tissues in the body. Common symptoms of SLE include fatigue, joint pain and swelling, skin rashes, sensitivity to sunlight, fever, chest pain, hair loss, and mouth sores.^15^ Symptoms can be diverse and affect different individuals differently. Diagnosing SLE can be challenging because it mimics other conditions. It often involves a combination of clinical evaluation, blood tests for autoantibodies and consideration of specific criteria established by medical organizations like the ACR. LN is a severe complication of SLE.^16^, ^17^, ^18^, ^19^, ^20^ In LN, the immune system mistakenly attacks the kidneys, leading to inflammation and damage. This condition can range from mild to severe and may cause symptoms such as proteinuria, hematuria, high blood pressure, and kidney dysfunction. LN can have a significant impact on the prognosis and overall health of individuals with SLE.

Currently, in clinical practice, the assessment of disease activity in LN has primarily relied on disease AI scoring, including BILAG or SLEDAI scoring systems, as well as biomarker‐based activity assessment.^21^ However, it is often challenging to determine whether positive indicators are caused by LN. Kidney biopsy, as an invasive examination, imposes significant trauma and risks to patients, and it cannot be repeated, thus having significant limitations. Serological markers, on the other hand, play a crucial role in diagnosing and differentiating various diseases in clinical practice due to their simplicity, speed, and high specificity.

IFN‐γ is a type of pro‐inflammatory cytokine primarily secreted by Th1 cells. It plays a crucial role in the pathogenesis of SLE and is closely associated with kidney damage in SLE. In individuals with SLE and LN, there is often an overactive immune system, and IFN‐γ is produced h1 cells, and can contribute to this immune dysregulation.^6^, ^22^, ^23^ It promotes inflammation and the production of autoantibodies, which are antibodies that mistakenly target the body's own tissues and organs. Therefore, IFN‐γ has been considered as a potential therapeutic target in the treatment of LN. Clinical trials and research are ongoing to explore the effectiveness of drugs that inhibit IFN‐γ or its downstream pathways in managing LN.

CXCL9,^16^ CXCL10, and CXCL11^24^, ^25^ are produced in response to IFN‐γ and are IFN‐γ‐inducible chemokines. They can bind to a common receptor CXCR3.^26^, ^27^, ^28^ This receptor is found on various immune cells, including T cells, natural killer (NK) cells, and dendritic cells. When these chemokines bind to CXCR3, they promote the migration of immune cells to the kidneys, contributing to renal inflammation, and cause the recruitment and activation of immune cells in the kidneys, and immune cell infiltration which contribute to renal inflammation and damage LN.^29^ However, no clear clinical application has been established yet.

Our findings suggested that serum CXCL9, CXCL10, and CXCL11 levels were significantly higher in SLE‐LN patients, comparing to SLE patients and HCs, and the ROC analysis of CXCL chemokines can significantly improve sensitivity and specificity for the diagnosis of LN in SLE patients, demonstrating the diagnostic value of serum CXCL9, CXCL10, and CXCL11 in LN and their correlation with the activity level of LN.

There are some shortcomings in this study. First, this study was a retrospective and single‐center study. Therefore, this result may have biases. Second, our results demonstrate there is a correlation between the serum levels of CXCL9, CXCL10, and CXCL11 and the activity level of LN in SLE patients with concurrent LN, while it is not clear whether their abnormal levels are specifically caused by LN activity. Third, it would be interesting to further study the effects and the molecular mechanisms underlying the participation of CXCL9, CXCL10, and CXCL11 in LN using an animal model. Fourth, BILAG scoring system could be employed in the future study instead of SLEDAI activity score. Last, all the reviewed cases were Han people, future studies could include other peoples and other races to verify the current findings.

## 5 CONCLUSIONS

Current study demonstrates that serum CXCL9, CXCL10, and CXCL11 levels can significantly improve sensitivity and specificity for the diagnosis of LN in SLE patients.

## AUTHOR CONTRIBUTIONS

**Shuo Wang**: Data curation; data analysis; drafting of the article; final approval of the version to be published.**Yanhui Cui**: Study supervision; coordination; funding support; design of this study; drafting of the article; final approval of the version to be published.

## CONFLICT OF INTEREST STATEMENT

The authors declare no conflicts of interest.

## ETHICS STATEMENT

This is a retrospective study and was approved by Zibo Central Hospital (#2023.05.284). Informed written consent from the participants was waived. The study was performed in strict accordance with the Declaration of Helsinki, Ethical Principles for Medical Research Involving Human Subjects. Current study is available from the corresponding author on reasonable request.

## Supporting information

Supporting information.

## Data Availability

The data could not be shared openly, as required by our department. The raw data could be obtained upon reasonable request to the corresponding author.
