# Supplementary material for: Clinical significance of serum CXCL9, CXCL10, and CXCL11 in patients with lupus nephritis
Source: Immun Inflamm Dis. 2024 Aug 22;12(8):e1368. doi: 10.1002/iid3.1368 (PMC11340012; doi:10.1002/iid3.1368)
Supplement: Supplementary file 1 — Supporting information. [file IID3-12-e1368-s001.docx]

**Supplementary materials**

Table S1. Spearman correlation analysis of serum CXCL9, CXCL10 with CXCL11 with systemic lupus erythematosus disease activity index (SLEDAI) in all systemic lupus erythematosus (SLE) patients (n = 160).

|  | SLEDAI | |
| --- | --- | --- |
|  | r | p value |
| Serum CXCL9 | 0.362 | < 0.001 |
| Serum CXCL10 | 0.416 | < 0.001 |
| Serum CXCL11 | 0.297 | < 0.001 |

Table S2. Spearman correlation analysis of serum CXCL9、CXCL10 and CXCL11 with systemic lupus erythematosus disease activity index (SLEDAI) in systemic lupus erythematosus patients without lupus nephritis (SLE, n = 92).

|  | SLEDAI | |
| --- | --- | --- |
|  | r | p value |
| Serum CXCL9 | 0.145 | 0.006 |
| Serum CXCL10 | 0.037 | 0.152 |
| Serum CXCL11 | 0.081 | 0.089 |
